# T Cells and Macrophages Responding to Oxidative Damage Cooperate in Pathogenesis of a Mouse Model of Age-Related Macular Degeneration

**DOI:** 10.1371/journal.pone.0088201

**Published:** 2014-02-19

**Authors:** Fernando Cruz-Guilloty, Ali M. Saeed, Stephanie Duffort, Marisol Cano, Katayoon B. Ebrahimi, Asha Ballmick, Yaohong Tan, Hua Wang, James M. Laird, Robert G. Salomon, James T. Handa, Victor L. Perez

**Affiliations:** 1 Bascom Palmer Eye Institute, Department of Ophthalmology, University of Miami Miller School of Medicine, Miami, Florida, United States of America; 2 Department of Microbiology and Immunology, University of Miami Miller School of Medicine, Miami, Florida, United States of America; 3 Wilmer Eye Institute, Department of Ophthalmology, Johns Hopkins University School of Medicine, Baltimore, Maryland, United States of America; 4 Department of Chemistry, Case Western Reserve University, Cleveland, Ohio, United States of America; University of Florida, United States of America

## Abstract

Age-related macular degeneration (AMD) is a major disease affecting central vision, but the pathogenic mechanisms are not fully understood. Using a mouse model, we examined the relationship of two factors implicated in AMD development: oxidative stress and the immune system. Carboxyethylpyrrole (CEP) is a lipid peroxidation product associated with AMD in humans and AMD-like pathology in mice. Previously, we demonstrated that CEP immunization leads to retinal infiltration of pro-inflammatory M1 macrophages before overt retinal degeneration. Here, we provide direct and indirect mechanisms for the effect of CEP on macrophages, and show for the first time that antigen-specific T cells play a leading role in AMD pathogenesis. *In vitro*, CEP directly induced M1 macrophage polarization and production of M1-related factors by retinal pigment epithelial (RPE) cells. *In vivo*, CEP eye injections in mice induced acute pro-inflammatory gene expression in the retina and human AMD eyes showed distinctively diffuse CEP immunolabeling within RPE cells. Importantly, interferon-gamma (IFN-γ) and interleukin-17 (IL-17)-producing CEP-specific T cells were identified *ex vivo* after CEP immunization and promoted M1 polarization in co-culture experiments. Finally, T cell immunosuppressive therapy inhibited CEP-mediated pathology. These data indicate that T cells and M1 macrophages activated by oxidative damage cooperate in AMD pathogenesis.

## Introduction

Age-related macular degeneration (AMD) is a complex and heterogeneous collection of retinal diseases representing the leading cause of blindness in industrialized countries [1], [2]. There are two major classifications of AMD: dry and wet AMD. Dry AMD is a result of dysfunctional retinal pigment epithelial (RPE) cells, which are in charge of nourishing and protecting the retina, especially the photoreceptor (PR) cells [3]. Clinical features of dry AMD include the presence of drusen (accumulated debris in the form of visible yellow spots), geographic atrophy (focal loss of the RPE layer), PR cell death and ultimately loss of central vision. In contrast, wet AMD is a more severe, end-stage form of the disease that occurs in ∼10% of total cases when blood vessels from the underlying choroid abnormally grow into the outer retina, a process called choroidal neovascularization (CNV) [4], [5]. Laser-induced CNV (although technically involving a wound healing response) serves as a valuable model of wet AMD in many species, and intraocular anti-VEGF therapy, which inhibits the angiogenesis associated with CNV, is useful for the treatment of wet AMD [6]. Unfortunately, there is no treatment for dry AMD and the pathogenic mechanisms remain to be fully elucidated. Recent evidence now implicates the immune system in the development of AMD [7], [8], as immune-related proteins are found in drusen from AMD eyes [9] and genome-wide association studies have linked specific polymorphisms in complement factor genes with the development of AMD [10]–[15]. In this light, it has been suggested that AMD can be viewed as a chronic inflammatory disease [16]–[19]. Thus, the detailed analysis of immune responses at the onset of disease opens the door for a greater understanding of AMD etiology mechanisms.

The immune system can be broadly divided into innate (general, non-specific immune system) and adaptive (specific) immunity. While many aspects of the interplay between innate and adaptive immune systems have been studied in the setting of acute bacterial or viral infections [20], [21], much less is known about their mechanistic crosstalk in the context of chronic inflammatory diseases, such as cancer, atherosclerosis and heart disease [22]. Information related to the AMD disease process could apply to chronic inflammation in general. Two relevant cell types that merit attention are macrophages and T cells. Macrophages are essential components of the innate immune system and have been the subject of close inspection in the context of AMD [23]–[29], although their specific roles at different stages of disease progression remain controversial. Macrophage differentiation is mostly dictated by the microenvironment and has profound implications for proper activation and function [30], [31]. Pro-inflammatory M1 macrophages produce tumor necrosis factor-alpha (TNF-α) and interleukin-12 (IL-12), and are associated with tissue destruction, whereas M2 macrophages, characterized by production of the immunosuppressive cytokine IL-10, play a role in tissue homeostasis and repair. On the other hand, T cells are major effector cells of the adaptive immune response, providing the antigen specificity required for proper immune responses. Two major classes of T cells include CD4+ helper T (Th) cells and CD8+ cytotoxic T lymphocytes (CTLs) [32], [33]. Th cells mainly shape the type of response based on the cytokines they release and help the recruitment and function of other immune cells, while CTLs are capable of directed killing of target cells. Our present knowledge regarding the role of T cells in AMD is surprisingly limited. Robert Nussenblatt and colleagues have shown that complement component 5a (C5a) induces the expression of IL-17 and IL-22 by human CD4+ T cells and that blood from AMD patients contains higher levels of these cytokines compared to controls [34]. Recently, AMD was also associated with age-related changes in peripheral T cells in humans, lending support to the idea that AMD can be a systemic disease [35]. However, the identity of antigen-specific T cells that potentially mediate AMD pathology and how they may interact with other immune cells (e.g. macrophages and B cells) in chronic retinal inflammation remains to be determined.

A potential link between innate and adaptive immune responses in disease is oxidative stress, a known contributing factor in the development of pathological inflammatory conditions, including atherosclerosis and AMD [36]. Lipid peroxidation has been shown to produce oxidation specific epitopes (OSEs) that can function as new antigens for immune recognition [37]. One of the best-characterized OSEs in the context of AMD is carboxyethylpyrrole (CEP) a protein adduct resulting from an oxidation fragment of docosahexaenoic acid (DHA) [38]. AMD donor eyes contain more CEP-modified proteins in the outer retina and drusen than age-matched controls [9], although the precise histological localization patterns of CEP within the healthy or diseased human retina have not been described. CEP-modified proteins and CEP autoantibodies are also more abundant in AMD plasma than in control samples [38], [39]. Our laboratory immunized mice with CEP-adducted mouse serum albumin (CEP-MSA) and produced a novel mouse model with dry AMD-like pathology [40], [41], effectively making the connection between an adaptive immune response (due to the presence of CEP-specific antibodies) and the onset of AMD. Furthermore, we recently showed that pro-inflammatory M1 macrophages are recruited to the retina of CEP-immunized mice prior to overt degeneration [26]. While the laser-induced CNV model of wet AMD has provided great insight into the disease process and potential therapies (especially in the context of angiogenesis), the CEP model is to date the only one available for the immunological study of the dry form of the disease in genetically unmanipulated animals.

Here we provide evidence that CEP serves as an initiating signal for the cooperation of innate and adaptive immunity in the pathogenesis of AMD. CEP acts directly and indirectly to influence M1 macrophage polarization. It can directly activate macrophages, leading to M1 gene expression. We also find CEP-specific T cells from CEP-immunized mice that produce the inflammatory cytokines interferon-gamma (IFN-γ) and IL-17 that are able to induce M1 macrophage polarization *in* *vitro*. Surprisingly, we observed CEP-mediated retinal pathology in mice lacking mature B cells, indicating that AMD-like pathology in our model is antibody-independent and T cell-mediated. Analysis of mice with defects in several T cell differentiation pathways suggests that Th1 (IFN-γ producing) cells are important for development of disease. Finally, pharmacological inhibition of T cell activation prevents retinal pathology in our model, providing proof of concept for the use of immunotherapy in AMD treatment. This study provides cellular and molecular mechanisms that explain the role of inflammation in AMD: M1 macrophages and antigen-specific T cells activated by oxidative damage-induced products work together at the early onset stage of dry AMD. Disruption of this cooperation could lead to innovative therapies for this highly prevalent disease.

## Materials and Methods

### Ethics Statement

Protocols for use of experimental animals in this study adhered to the ARVO Statement for the Use of Animals in Ophthalmic and Vision Research and were approved by the Institutional Animal Care and Use Committee of the University of Miami Miller School of Medicine (Protocol 11–321). All surgical procedures were performed under anesthesia and all efforts were made to minimize suffering.

### Mice

The following mice were obtained from The Jackson Laboratory: BALB/cJ (stock #000651) wild type mice, C57BL/6J (stock #000664) wild type mice, *μMT−/− (B6.129S2-Ighm<tm1Cgn>/J, stock #002288)*, *Stat6−/− (BALB/c background, C.129S2-Stat6<tm1Gru>/J, stock #002828),* and *Tbx21−/− (B6.129S6-Tbx21<tm1Glm>/J, stock #004648)*. *Tlr2−/− (B6)* mice were kindly provided by Dr. Dmitry Ivanov, along with corresponding littermate controls (Bascom Palmer Eye Institute). *Il17a−/− (BALB/c)* mice were kindly provided by Dr. Abul Abbas (UCSF). All mice were housed in a room exposed to 300 lux (outside the cage) in a 12 hr dark/light cycle.

### Antigen

CEP-MSA was prepared from commercially available mouse serum albumin (Sigma-Aldrich), which was converted to CEP-modified MSA following previously published procedures [42].

### Immunizations

The CEP-MSA immunization protocol has been described previously [40]. In summary, mice were primed by hind leg injections of 200 µg CEP-MSA in complete Freund’s adjuvant (CFA; from DIFCO) at 8–12 weeks of age. At day 10 post-immunization (p.i.), the mice were challenged in the neck with 100 µg CEP-MSA in incomplete Freund’s adjuvant (IFA; from DIFCO), followed by a final boost with 100 µg CEP-MSA in CFA in the neck seven days before harvest. As described in Cruz-Guilloty et al., 2013 [26], mice harvested at 40–90 days p.i. were defined as early recovery times, those harvested at 100–200 days p.i. were defined as intermediate recovery times, while those harvested after day 200 p.i. were considered late recovery times. Anti-CEP antibody titers at days 40–60 p.i. were quantified by ELISA as previously described [40] and used to determine efficiency of immunization. All immunized mice were compared with age-matched naïve, sham-MSA or CFA only controls. As previously reported [26], [40], there are no significant differences among the control mice (with low to undetectable anti-CEP titers) in terms of retinal pathology and are therefore used interchangeably, depending on experimental setup, and collectively labeled as “control”. For each mouse strain that was analyzed in this study, 2 or 3 independent experiments were performed with n = 3–5 mice per group per time point.

### Histology

Eyes were harvested at early (40–90 days), intermediate (100–200 days) and late (over 200 days) recovery times post-immunization (p.i.). Right eyes were used for histology and were fixed in 2% paraformaldehyde and 2.5% glutaraldehyde in 0.1M PO_4_ buffer (pH = 7.4) overnight and dehydrated in graded ethanol and propylene oxide. After polymerization in a resin mixture containing Polybed 812 (Polysciences) and Araldite 502 (Polysciences), semi-thin (0.7 µm) sagittal sections of each eye were stained with toluidine blue and analyzed for histopathology with light microscopy using a Zeiss microscope (equipped with an AxioCam digital camera) using a 63x oil-immersion lens.

### Pathology Scoring: Quantification of Lesions and Inflammatory Cells in the IPM

Each individual mouse in this study was scored for retinal pathology on a masked fashion, using 10 sections of the right eye with at least 25–30 µm intervals between each section. Scoring was divided in 2 subclasses: 1) the retinal lesion count represents the sum of RPE areas showing abnormal vesiculation, swelling, thinning, pyknosis, and cell lysis; 2) inflammatory cells were defined as dark nuclear stains of macrophage-like cells observed and counted only within the interphotoreceptor matrix (IPM) compartment at the level of the photoreceptor outer segments and the apical border of the RPE. The overall pathology score for each eye is the sum of the two subclasses. The data is always presented as pathology (cells and lesions combined) per section unless specified otherwise. A total of 3–5 mice were used in the analysis at each time point. At least two independent experiments were performed for each strain reported in this study. Repeat experiments with similar results were analyzed separately because of the use of independent batches of CEP-MSA.

### Immunohistochemistry

Identification of inflammatory cells in the mouse retinas was performed as previously described (Cruz-Guilloty et al, 2013).

The following was the protocol for analysis of human eyes. Autopsy eyes (n = 10) were obtained from the Wilmer Eye Institute Pathology Division after approval from the Human Subjects Committee at Johns Hopkins University. “Unaffected” eyes (n = 5) had no AMD history or microscopic evidence of drusen, basal deposits, or loss of RPE cuboidal epithelial morphology. Early AMD donors (n = 5) had an AMD history and macular drusen, but no late stage disease. Eyes were fixed in 4% formaldehyde, paraffin embedded, sectioned to 4 µm thickness, and deparaffinized with xylene and an ethanol gradient. Antigens were retrieved with the Target Retrieval System (Dako, Inc., Carpinteria, CA). Sections were incubated with blocking serum for 1 h; with mouse monoclonal anti-CEP antibody (30 µg/ml) or equivalent concentrations of mouse IgG1 isotype control overnight at 4°C; with biotinylated anti-mouse IgG for 60 min, and then with ABC-AP (Vector labs, Burlingame, CA) for 30 min. The chromagen was developed with blue substrate working solution (Vector labs) supplemented with levamasole. The IgG1 monoclonal anti-CEP antibody described here was produced by our lab in conjunction with Genscript, Inc. (Piscataway, NJ) following our standard immunization protocol and was tested (by ELISA and Western blots) for specificity against a variety of CEP-modified proteins and lacked recognition of other lipid-derived modifications (data not shown).

### Enzyme-linked Immunosorbent Assay (ELISA) for the Quantification of Cytokines in Culture and Serum

ELISAs for cytokine detection were performed, as per manufacturer’s instructions, using paired antibodies against IFN-γ, IL-17A, IL-2, IL-4, IL-1β, IL-12, TNF-α and IL-10 (all purchased from eBioscience, San Diego, CA). Purified recombinant cytokines from the same vendor were used to develop standard curves. In summary, flat bottom, high binding 96-well plates were coated with 100 µl of primary (capture) antibody in PBS, and washed with 150 µl of washing solution (PBS, 0.1% Tween-20). Blocking was done with 100 µl of the dilution buffer (PBS, 0.1% Tween-20, 1% BSA) followed by additional washes before adding 100 µl per well of protein (purified cytokine) standards or samples (undiluted CEP culture supernatants or 1∶25 serum dilution). After incubation and washing, 100 µl per well of secondary (biotin-labeled) antibody were added, followed by addition of 50 µl per well of Avidin-Alkaline Phosphatase (Sigma-Aldrich). After incubation, 50 µl of developing solution (Phosphatase Substrate from Sigma-Aldrich in DEA buffer at 1 mg/ml) were loaded per well and the absorbance was measured in a microplate reader at 405 nm.

### Gene Expression Analysis of Acute Retinal Inflammation by Quantitative Real Time PCR (RT-PCR)

Intravitreal injections were performed as follows. C57BL/6j mice (male and female, 2 months old) were anesthetized and the pupils dilated. Using a dissecting microscope, intravitreous injections into one eye of each mouse (n = 5 per group) were performed with a pump microinjection apparatus (Harvard Apparatus, Holliston, MA) and a glass micropipette that was calibrated to deliver 1 µl of vehicle containing either BSA (2 µg), CEP-BSA (2 µg), MSA (2.44 µg), or CEP-MSA (2.44 µg) on depression of the foot switch. Mice were sacrificed 6 and 24 hours later, eyes were enucleated, and the RPE/choroid and retinas were dissected. Total RNA was extracted using the RNeasy mini kit (Qiagen, Valencia, CA) according to the manufacturers protocol, and reverse transcription was perform using the High Capicity RNA-to-cDNA kit (Life Technologies, Grand Island, NY) following the manufacturer’s protocol. Analyses of selected genes were performed with TaqMan Gene Expression Assays (Life Technologies, Grand Island, NY), using the StepOnePlus TaqMan System Fast Mode (Life Technologies, Grand Island, NY). Data were analyzed using the comparative CT method. Primer and probe sets were as follows: *IL-1β*, Mm01336189_m1; *TNF-α*, Mm00443258_m1; *IL-6*, Mm00446190_m1; *IL-12,* Mm00434165_m1*; Ccl2*, Mm00441242_m1; *IL-10*, Mm00439614_m1; *KC*, Mm 04207460_m1.

### *In vitro* Culture and Stimulation of Primary Macrophages, RPE Cells and Splenocytes (CEP-specific T cells)

For *in* *vitro* macrophage cultures, bone marrow-derived macrophages (BMDM) were used. Bone marrow cells were differentiated in DMEM (Invitrogen) supplemented with 10% heat-inactivated FBS (Atlanta Biologicals) and 15% L929 cell-conditioned media (as a source of M-CSF) for seven days. Macrophage culture purity was determined by flow cytometry and cultures were >97% double positive for F4/80 and CD11b. Macrophages were stimulated in fresh DMEM with CEP-MSA (100 µg/ml), sham-MSA (100 µg/ml), or left untreated for 4–24 hours. The CEP-MSA and sham-MSA preparations were tested for endotoxin contamination using the ToxinSensor Chromogenic LAL Endotoxin Assay Kit (GenScript) and endotoxin levels were determined to be below 1 endotoxin unit (EU)/mL, with similar levels in both solutions. Lipopolysaccharide (LPS; from Sigma) at 100 ng/ml and Pam3CSK4 (InvivoGen) at 500 ng/ml were used as controls. After stimulation, the cells were used for RNA extraction (4–6 hr time point), while the supernatant was used for detection of secreted factors (24 hr time point). For detection of secreted proteins (TNF-α, IL-12 and IL-1β), ELISAs were performed as described above using antibody pairs and recombinant protein standard curves as per manufacturer’s instructions (eBioscience).

For *in* *vitro* RPE cultures, eyes were enucleated from mice and bisected along the ora serrata. The anterior portions (cornea, lens, etc.) were discarded and the neuronal retina was carefully removed and separated from the posterior eyecups. Next, the posterior eyecups were incubated in 0.25% trypsin-EDTA (Invitrogen) at 37°C for 1 h. Then, the RPE cells were removed from the posterior eye cups, pelleted and washed with DMEM/F12 (Invitrogen) containing 20% heat-inactivated FBS (Atlanta Biologicals). RPE cells were seeded into 12-well culture plates in DMEM/F12 containing 20% heat-inactivated FBS and cultured for 4–8 weeks. RPE cells were stimulated in fresh DMEM with CEP-MSA (100 µg/ml), sham-MSA (100 µg/ml), or left untreated for 4 hours.

To isolate CEP-specific T cells, splenocytes from mice immunized with CEP-MSA (or control mice) were stimulated *in* *vitro* with 100 µg/ml of antigen (CEP-MSA or Sham-MSA) in complete DMEM media. After 4 days, cultures were divided in two, with recombinant human IL-2 (20 units/ml; eBioscience) added to one of the two samples, followed by two more days of incubation. Flow cytometry acquisition (FACS Diva software on LSR-II cytometer) and analysis (FlowJo software) were performed at different time points using antibodies specific for CD3, CD4, CD8, B220, CD19, CD11b, NK1.1, CD69, F4/80 (all from eBioscience). For T cell-macrophage co-culture experiments, CEP-specific T cells and naïve BMDM were co-cultured at different ratios and different time points (6, 12, 24 and 48 hr). Data shown here were obtained from the optimal conditions of 1∶1 T cell-BMDM ratio and 24 hr co-culture period. The supernatants were collected to measure cytokine production by ELISA. For RT-PCR analysis of macrophage gene expression (as described below), T cells were removed from the cultures by vigorous pipetting and extensive washes in PBS (5 times), considering that BMDM are extremely adherent cells. Purity of the BMDM (∼95% F4/80+ cells) after co-culture was confirmed by flow cytometry.

### Gene Expression Analysis of BMDM by Quantitative Real Time PCR (RT-PCR)

Total RNA was isolated from cells using the RNeasy kit (Qiagen), then cDNA was generated with the Maxima First Strand cDNA Synthesis Kit (Fermentas). Gene expression was measured by real-time quantitative PCR (qPCR) using various primers (see below). qPCR was performed using iQ SYBR Green Supermix (Bio-Rad) on a Roche Light Cycler real-time PCR instrument. Relative gene expression was calculated using the ΔΔC_t_ method, with gene expression normalized to Gapdh expression. Each treatment is represented as relative-expression (i.e., fold-expression over reference group), where the control sample served as the reference with a set value of 1.

The following primer sets were used:

iNOS (**For:** GTTCTCAGCCCAACAATACAAGA, **Rev:** GTGGACGGGTCGATGTCAC).

TNF-α (**For:** CTGAACTTCGGGGTGATCGG, **Rev:** GGCTTGTCACTCGAATTTTGAGA).

IL-12A (**For:** CAATCACGCTACCTCCTCTTTT,

**Rev:** CAGCAGTGCAGGAATAATGTTTC).

IL-1b (**For:** GCAACTGTTCCTGAACTCAACT, **Rev:** ATCTTTTGGGGTCCGTCAACT).

IL-10 (**For:** GCTCTTACTGACTGGCATGAG, **Rev:** CGCAGCTCTAGGAGCATGTG).

Arginase-1 (**For:** CTCCAAGCCAAAGTCCTTAGAG,

**Rev:** AGGAGCTGTCATTAGGGACATC).

HO-1 (**For:** AAGCCGAGAATGCTGAGTTCA, **Rev:** GCCGTGTAGATATGGTACAAGGA).

Srxn1 (**For:** CCCAGGGTGGCGACTACTA, **Rev:** GTGGACCTCACGAGCTTGG).

Ccl2 (**For:** TTAAAAACCTGGATCGGAACCAA,

**Rev:** GCATTAGCTTCAGATTTACGGGT).

IL-6 (**For:** CTGCAAGAGACTTCCATCCAG, **Rev:** AGTGGTATAGACAGGTCTGTTGG).

KC (**For:** ACTGCACCCAAACCGAAGTC, **Rev:** TGGGGACACCTTTTAGCATCTT).

Vegf-A (**For:** GCACATAGAGAGAATGAGCTTCC, **Rev:** CTCCGCTCTGAACAAGGCT).

Vegf-B (**For:** GCCAGACAGGGTTGCCATAC, **Rev:** GGAGTGGGATGGATGATGTCAG).

Gapdh (**For:** AGGTCGGTGTGAACGGATTTG, **Rev:** TGTAGACCATGTAGTTGAGGTCA).

### Animal Treatment with the Immunosuppressive Drugs Cyclosporine A and Rapamycin

Wild-type (8-week old) BALB/cJ mice were immunized with CEP-MSA in CFA and treated with a combination therapy of cyclosporine A (CsA; “Sandimmune” from Novartis, East Hanover, NJ) and rapamycin (Rapa; “Rapamune” from Wyeth Pharmaceuticals, Philadelphia, PA) with daily intraperitoneal (i.p.) injections for 21 or 40 days (starting on the first day of immunization). Blood was collected at day 50 p.i. for anti-CEP titer evaluation and eyes were harvested at day 60–100 p.i. (early recovery time) for histological analysis. The stock solutions where diluted in sterile PBS for 100 µl i.p. injections at the following doses: CsA = 5 mg/kg/day; Rapa = 0.5 mg/kg/day.

### Statistics

Results shown in graphs are presented as means +/− S.D. Data for direct comparison of two samples were analyzed using two-tailed Student’s t test with Prism software (GraphPad). Analyses for 3 groups or more were performed using one-way analysis of variance (ANOVA) followed by Newman-Keuls multiple comparison tests, also with Prism software. P-values <0.05 were considered significant.

## Results

### CEP Directly Activates Macrophages, Leading to M1 Polarization *In vitro*

**Figure 1:**
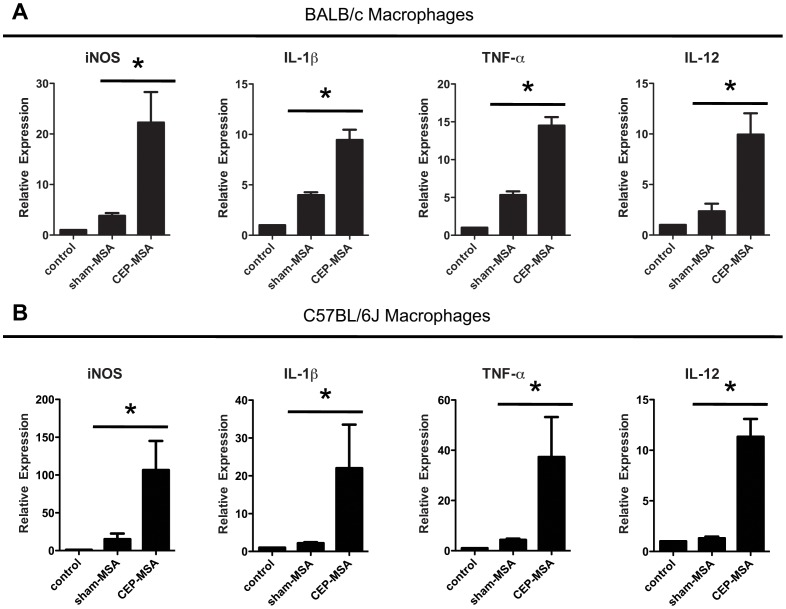
CEP directly activates macrophages *in* *vitro*, leading to M1 polarization. (**A**) Bone marrow-derived macrophages (BMDM) from BALB/c mice were stimulated for 4 hrs with CEP-MSA (100 µg/ml), Sham-MSA (100 µg/ml) or left untreated. RNA was isolated and qPCR was used for gene expression analysis. Each treatment is represented as relative-expression (i.e., fold-expression over reference group), where the control (untreated) sample served as the reference with a set value of 1. CEP specifically induced the expression of M1 markers (iNOS, IL-1β, TNF-α and IL-12A) but had no effect on M2 or Mox marker genes (Figure S1). (**B**) BMDM from C57BL/6 mice were stimulated and analyzed as described above. Data from 4 independent experiments were pooled and two-tailed Student’s t tests were used for statistical analysis (* denotes p<0.05); error bars represent S.D.

**Figure 2:**
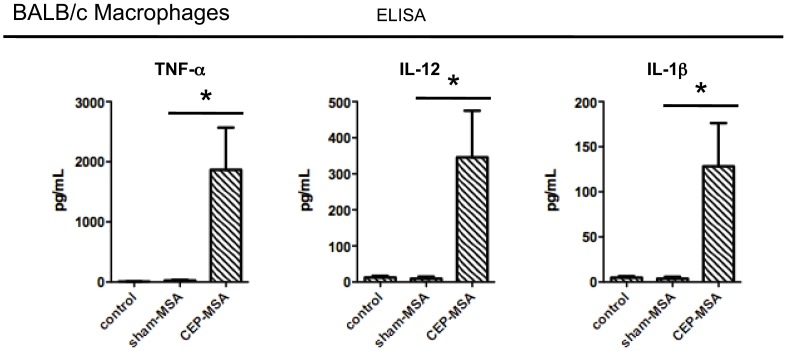
CEP induces secretion of M1-type cytokines by activated macrophages. Bone marrow-derived macrophages from BALB/c mice were stimulated for 24 hrs with CEP-MSA (100 µg/ml), Sham-MSA (100 µg/ml) or left untreated. Supernatants were collected and used for detection of secreted proteins by ELISA. CEP specifically induced the secretion of M1 cytokines (TNF-α, IL-12 and L-1β). Data from at least 2 independent experiments were pooled and ANOVA was used for statistical analysis (* denotes p<0.05); error bars represent S.D.

We recently reported that CEP immunization in mice leads to Ccl2/Ccr2-mediated M1 macrophage recruitment to the outer retina before AMD-like pathology ensues [26]. However, the mechanisms by which CEP promotes M1 polarization remained to be elucidated. Because oxidized phospholipids and their resulting protein modifications have been shown to activate macrophages [29], [43], we tested whether CEP has a direct effect on macrophage activation using an *in* *vitro* system. Bone-marrow derived macrophages (BMDM) were differentiated *in* *vitro* and stimulated with CEP-MSA, Sham-MSA (non-adducted MSA) or left untreated, followed by quantitative PCR (qPCR) analysis of gene expression. We used two different wild type strains (BALB/c and C57BL/6J), since we have shown that both develop CEP-mediated pathology [26], [40]. As shown in **Fig. 1**, CEP-MSA specifically induced the expression of M1 marker genes (iNOS, IL-1β, TNF-α, IL-12) in both BALB/c (**Fig. 1A**) and C57BL/6J (**Fig. 1B** **)** macrophages, but did not have a significant effect on the expression of M2 markers (Arg-1, IL-10) or genes (HO-1, Srxn1) expressed in a recently described class of macrophages activated by oxidized phospholipids termed Mox cells [43] (Figure S1). In addition to the M1 marker genes mentioned above, CEP induced macrophage expression of other pro-inflammatory cytokines such as IL-6 and KC, but did not have an effect on the pro-angiogenic genes Vegf-A and Vegf-B (Figure S2). Furthermore, the CEP-induced M1 polarization was confirmed at the protein level, as stimulated cells secreted IL-1β, TNF-α, and IL-12 (**Fig. 2**).

### CEP Promotes Acute Inflammatory Responses in RPE Cells *In vivo* and *In vitro*

**Figure 3:**
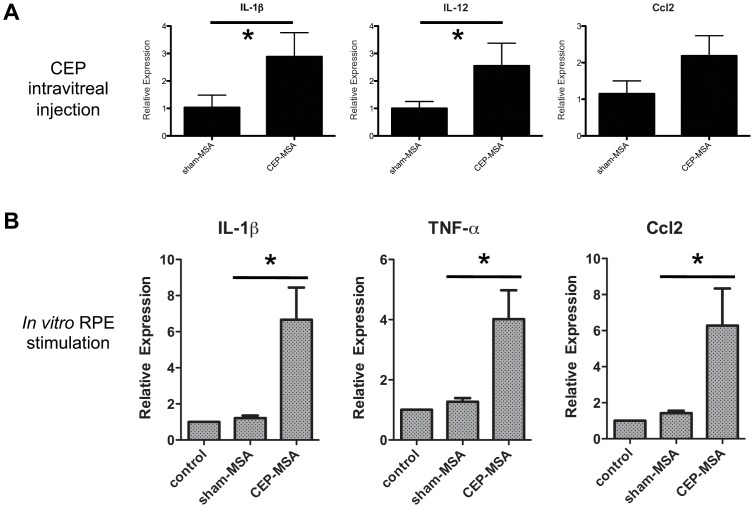
CEP induces pro-inflammatory gene expression in RPE cells *in* *vivo* and *in* *vitro*. (**A**) Intravitreal injections of CEP-MSA or Sham-MSA (2 µg total) were performed, RNA was isolated after 6 hrs from the RPE/choroid, followed by Taqman gene expression analysis (n = 5). Mean values from one of two independent experiments are shown; error bars represent S.D. (*) denotes statistically significant differences (p<0.05) based on two-tailed Student’s t tests. (**B**) Primary mouse RPE cultures were stimulated for 4 hrs with CEP-MSA (100 µg/ml), Sham-MSA (100 µg/ml) or left untreated. RNA was isolated and qPCR was used for gene expression analysis. Each treatment is represented as relative-expression (i.e., fold-expression over reference group), where the control (untreated) sample served as the reference with a set value of 1. Data are pooled from at least 2 independent experiments and include RPE cells from both BALB/c and B6 mice. Mean values from a total of four experiments are shown; error bars represent S.D. (*) denotes statistically significant differences (p<0.05) based on two-tailed Student’s t tests.

As previously described [40], systemic CEP immunization leads to a chronic, low-grade inflammatory response in the mouse retina, but experimental evidence for its *in situ* effects has not been reported to date. This is particularly relevant for the effects of CEP on the RPE, the cell type mostly affected in AMD. To further probe the ability of CEP to induce pro-inflammatory conditions in the retina, we performed intravitreal injections of CEP-MSA or Sham-MSA, followed by gene expression analysis of M1-associated transcripts in RPE/choroid samples isolated 6 hrs post-injection. As shown in **Fig. 3A**, injection of CEP-MSA (but not Sham-MSA) specifically induced the expression of a subset of M1-related genes (such as IL-1β, IL-12 and Ccl2), but not others (such as TNF-α, Figure S3). In addition, *in* *vitro* CEP stimulation of primary RPE cells (in the absence of choroid) also led to pro-inflammatory gene expression, including the production of the monocyte chemoattractant Ccl2 (**Fig. 3B**). Taken together, these data show that CEP can indeed promote a retinal microenvironment conducive to the recruitment of circulating monocytes (through the Ccl2/Ccr2 axis) and M1 polarization.

### CEP is Distinctively Localized within the RPE of Human AMD Eyes, but not in Healthy Eyes

**Figure 4:**
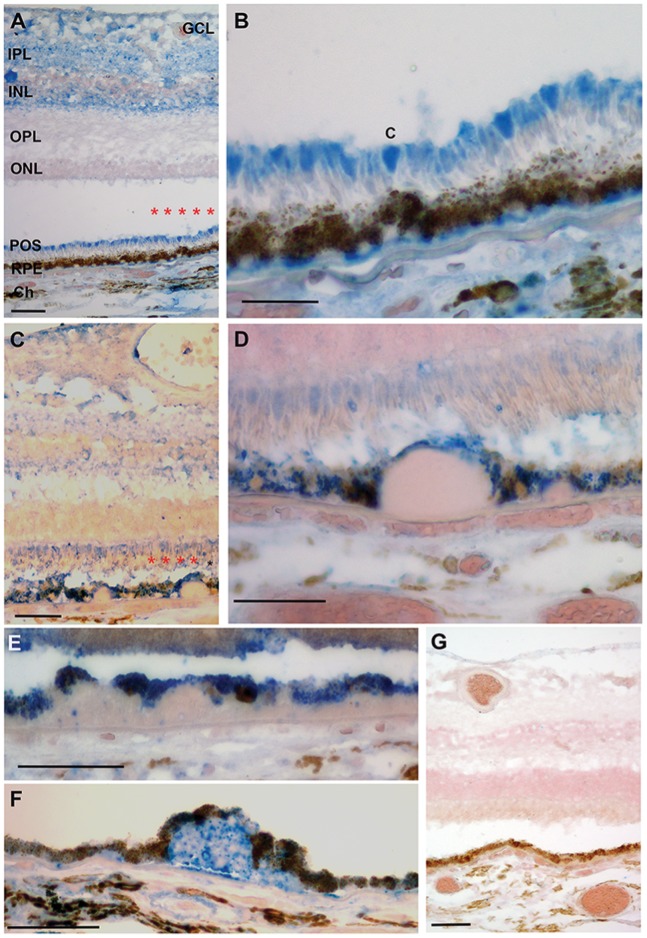
CEP immunolabeling in normal healthy eyes and dry AMD eyes. (**A**) Macula from a 77 yo unaffected female with CEP immunolabeling (blue) in the ganglion cell layer (GCL), inner plexiform layer (IPL), inner nuclear layer (INL), and photoreceptor outer segments (POS). Minimal labeling is seen in the RPE. Thin immunolabeling is seen at the RPE basement membrane. (**B**) Magnified view of the region labeled with red asterisks in A. POS are prominently labeled, especially cones (c). CEP immunolabeling at the basal RPE in inner Bruch’s membrane is more obvious. (**C**) Macula from a 51 yo male with dry (non-neovascular) AMD with CEP immunolabeling in the nerve fiber layer and GCL, IPL, INL, and photoreceptors. Labeling in photoreceptors is more prominent in the inner regions than in the POS. The RPE is diffusely labeled. (**D**) Magnified view of the region labeled with red asterisks in C shows diffusely labeled RPE. A prominent drusen is minimally labeled for CEP. (**E**) 69 yo female with non-neovascular AMD. The RPE cells overlying a large drusen are diffusely labeled for CEP. The drusen has a speckled labeling pattern for CEP. (**F**) 83 yo female with dry AMD. The drusen has prominent CEP immunolabeling with diffuse labeling within the overlying RPE. (**G**) IgG control from a 61 yo male with non-neovascular AMD. Ch, choroid; Bar = 25 µm.

Our CEP mouse model of dry AMD is based on observations from human AMD patients that show elevated levels of CEP in drusen and CEP autoantibodies [9], [38]. However, a detailed description of the CEP localization patterns in normal versus dry (non-neovascular) AMD retinas is still lacking. We took advantage of a new mouse IgG1 monoclonal antibody against CEP developed in our laboratory to perform CEP immunolabeling in human retinas (**Fig. 4**). Distinct differences in CEP immunolabeling were observed between maculas of unaffected control (n = 5; average age 85.4 yrs) and dry (non-neovascular) AMD donors (n = 5; average age 73.8 yrs). To our surprise, CEP was identified in unaffected control donor maculas, including the ganglion cell layer, inner plexiform layer, inner nuclear layer, and photoreceptor outer segments (**Fig. 4A–B**). Within the PR cells, CEP was more prominent in cones relative to rods. The RPE had minimal, if any, CEP labeling. When present, CEP was seen in inner Bruch’s membrane. Minimal labeling for CEP was present in the choroid. Like unaffected controls, CEP was identified in the inner retinal layers of AMD maculas, including the ganglion cell layer, inner plexiform layer, and inner nuclear layer (**Fig. 4C**). However, CEP immunolabeling was more prominent in the inner aspect of photoreceptors compared to outer segments. In addition, immunolabeling for CEP was diffusely seen within RPE cells of dry AMD eyes (**Fig. 4C–G**). CEP immunolabeling within drusen ranged from absent to prominent labeling. The presence of CEP within RPE of dry AMD patients complements our data on CEP-induced retinal inflammation in mice by suggesting that CEP signaling in the RPE associates with AMD pathology.

### CEP-induced Retinal Pathology is Antibody-independent and T Cell-mediated

**Figure 5:**
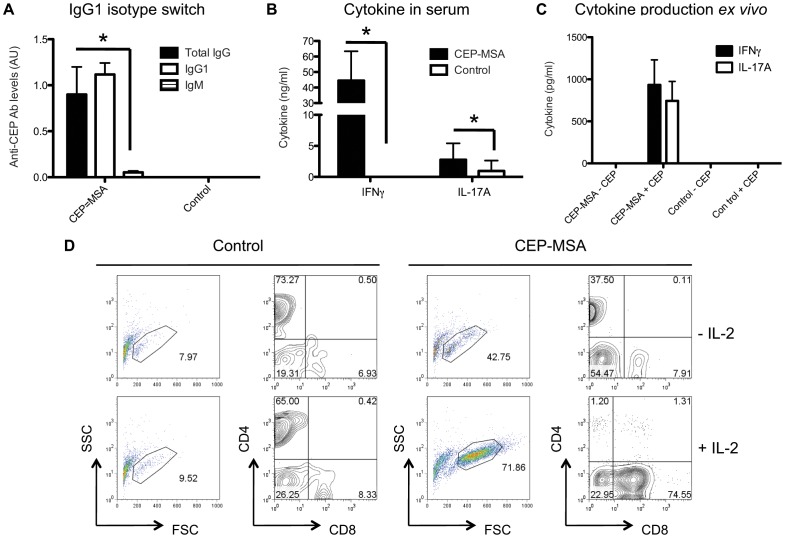
IgG1 isotype switch, pro-inflammatory cytokine production and CEP-specific T cell priming in CEP-MSA immunized mice. (**A**) Significant amounts of anti-CEP antibody titers are detected in serum from immunized mice. Isotype switch to IgG1 from IgM (undetectable levels) was observed. Naïve controls show undetectable levels of anti-CEP titers. ANOVA was used for statistical analysis (* denotes p<0.05). (**B**) Serum levels of IFN-γ and IL-17A were measured by ELISA. While both cytokines were present at higher levels in serum from CEP-immunized mice compared to control (naïve) mice, IFN-γ levels were predominantly elevated. Two-tailed Student’s t test was used for statistical analysis (* denotes p<0.05). (**C**) T cells from CEP-MSA immunized, but not naïve, mice produce IFN-γ and IL-17A when stimulated *in* *vitro* with the CEP-MSA antigen. (**D**) Splenocytes from WT B6 mice immunized with either CEP-MSA or CFA only were stimulated in vitro with CEP-MSA for 6 days *ex vivo*. At day 4 the cultures were divided in two, and IL-2 was added to the indicated samples. Cells were stained with anti-CD4 and anti-CD8 and analyzed by flow cytometry. Forward and side scatters are shown on the left with a “live lymphocyte” gate, CD8-CD4 plots show cells within this gate. Results shown here are mean values representative of more than 5 independent experiments for C57BL/6 mice (with n>3 for each experiment); error bars represent S.D. Similar results are observed with BALB/c mice (not shown).

Besides the apparent deleterious role of innate cells (M1 macrophages) in our CEP model of dry AMD [26], we have already published evidence for the role of the adaptive immune system. Not only does CEP immunization result in elevated titers of anti-CEP autoantibodies in wild type mice, but the same immunization protocol in RAG−/− mice (which lack mature T and B cells) fails to produce retinal pathology [40]. We now provide evidence for a leading role of antigen-specific T cells in the immune response against CEP. We observed specific antibody isotype switching (indicative of a T cell-dependent humoral response) from IgM (which was detected early at day 20 post-immunization, data not shown) to IgG1 (**Fig. 5A**), but not IgG2a or IgG2b (data not shown), in CEP immunized mice on both C57BL/6 and BALB/c backgrounds. Anti-CEP antibody production requires complete Freund’s adjuvant (CFA), as other adjuvants, such as Alum, fail to induce high titers (Figure S4). Because T cells are known for their ability to secrete cytokines, we quantified several types of cytokines in serum by ELISA and found elevated levels of the pro-inflammatory cytokines IFN-γ (44.5 ng/ml vs undetectable) and IL-17A (2.8 ng/ml vs 0.97 ng/ml) in serum of CEP-immunized compared to control mice (**Fig. 5B**). In addition, we observed a similar pattern of cytokine production *ex vivo* when splenocytes from CEP-immunized or control mice were stimulated with the cognate antigen (**Fig. 5C**). Production of the Th2-type cytokine IL-4 was not detected in these cultures (data not shown). To confirm the presence of CEP-specific T cells, we performed flow cytometric analysis of the *in* *vitro* stimulated splenocytes and found a clear population of activated T cells (gate in forward and side scatter plots) when cells from CEP-immunized mice (but not control mice such as mice immunized with CFA only) were cultured in the presence of CEP-MSA (**Fig. 5D**). T cell activation was absent in control conditions with Sham-MSA, irrelevant antigen (ovalbumin) and media only (data not shown). Both CD4+ and CD8+ T cells were present in the CEP-specific cultures, with increased expansion of the CD8+ population when IL-2 (a T cell growth and differentiation factor) was added to the cultures, most likely due to the known increased sensitivity for IL-2 and proliferation ability of CD8+ CTLs [44].

**Figure 6:**
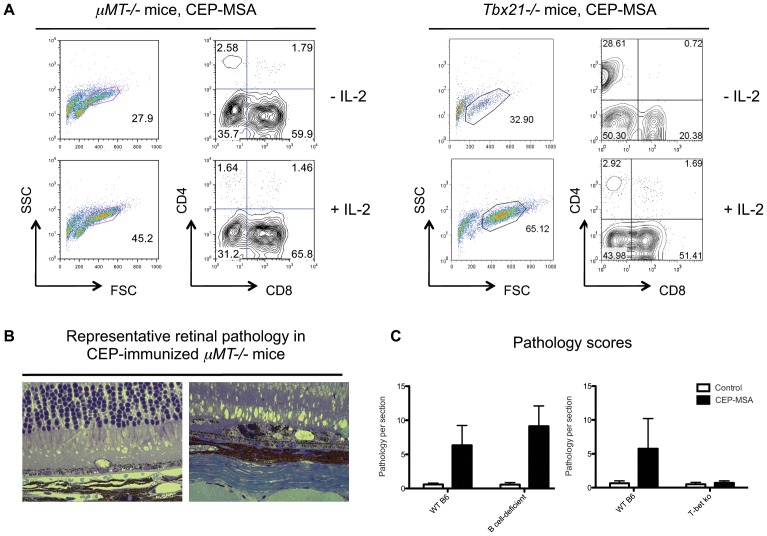
CEP-induced retinal pathology is antibody-independent and Th1 cell-mediated. (**A**) B cell deficient (*μMT−/−)* or T-bet deficient (*Tbx21−/−*) mice were immunized with CEP-MSA and splenocytes were activated *ex vivo* with CEP-MSA as described above. Forward and side scatters are shown on the left with a “live lymphocyte” gate, CD8-CD4 plots show cells within this gate. CEP-specific T cells were observed in the cultures for both strains. (**B**) CEP-MSA immunized *μMT−/−* mice develop AMD-like pathology. Pictures are representative of histological analysis using plastic sections as described in Ref. 24. (**C**) Retinal pathology scores for the indicated groups at the late time point (day 200+ p.i.; n = 3). Mean values are shown; error bars represent S.D. Data from one representative experiment for each strain were used for this analysis; similar results were obtained in at least one separate independent experiment per strain.

To formally test the role of antibody-producing B cells in our model, we immunized mice lacking only mature B cells (*μMT−/−* mice) with CEP-MSA (**Fig. 6**). While, as expected, anti-CEP antibody titers were not detectable in CEP-immunized *μMT−/−* mice (data not shown), CEP-specific T cell activation was observed, with most cells being CD8+ T cells (**Fig. 6A,** left panel). This result proves that T cells can be activated in the absence of B cells, which also function as antigen presenting cells (APCs). Importantly, CEP-mediated retinal pathology was observed in *μMT−/−* mice (**Fig. 6B** **,** quantification in **Fig. 6C,** left panel), indicating that AMD-like pathology in our model does not require antibodies and can be mediated by T cells only. To dissect which pathways of T cell differentiation may be more relevant to CEP-induced pathology, we immunized several knockout (ko) mice with specific defects in Th1, Th2 and Th17 responses. Anti-CEP antibody titers were not significantly affected in any of the tested ko mice (data not shown). Mice with impaired Th2 differentiation (Stat6 ko mice) or lacking IL-17A production (IL-17A ko mice) developed CEP-mediated pathology similar to WT mice (all on the BALB/c background; data now shown). However, T-bet ko mice (on the B6 background), with known defects in Th1 differentiation and IFN-γ production, did not develop retinal pathology after CEP immunization (**Fig. 6C**, right panel), even though CEP-specific T cells with increased IL-17A production were detected *ex vivo* (**Fig. 6A**, right panel and data now shown). Taken together, these results indicate that T cells, not B cells or antibodies, lead the adaptive immune response to CEP and are required for development of disease. Furthermore, Th1 differentiation (or IFN-γ production in general) seems to play an essential role in this process.

### CEP-specific T Cells Induce M1 Macrophage Polarization in Co-culture Experiments

**Figure 7:**
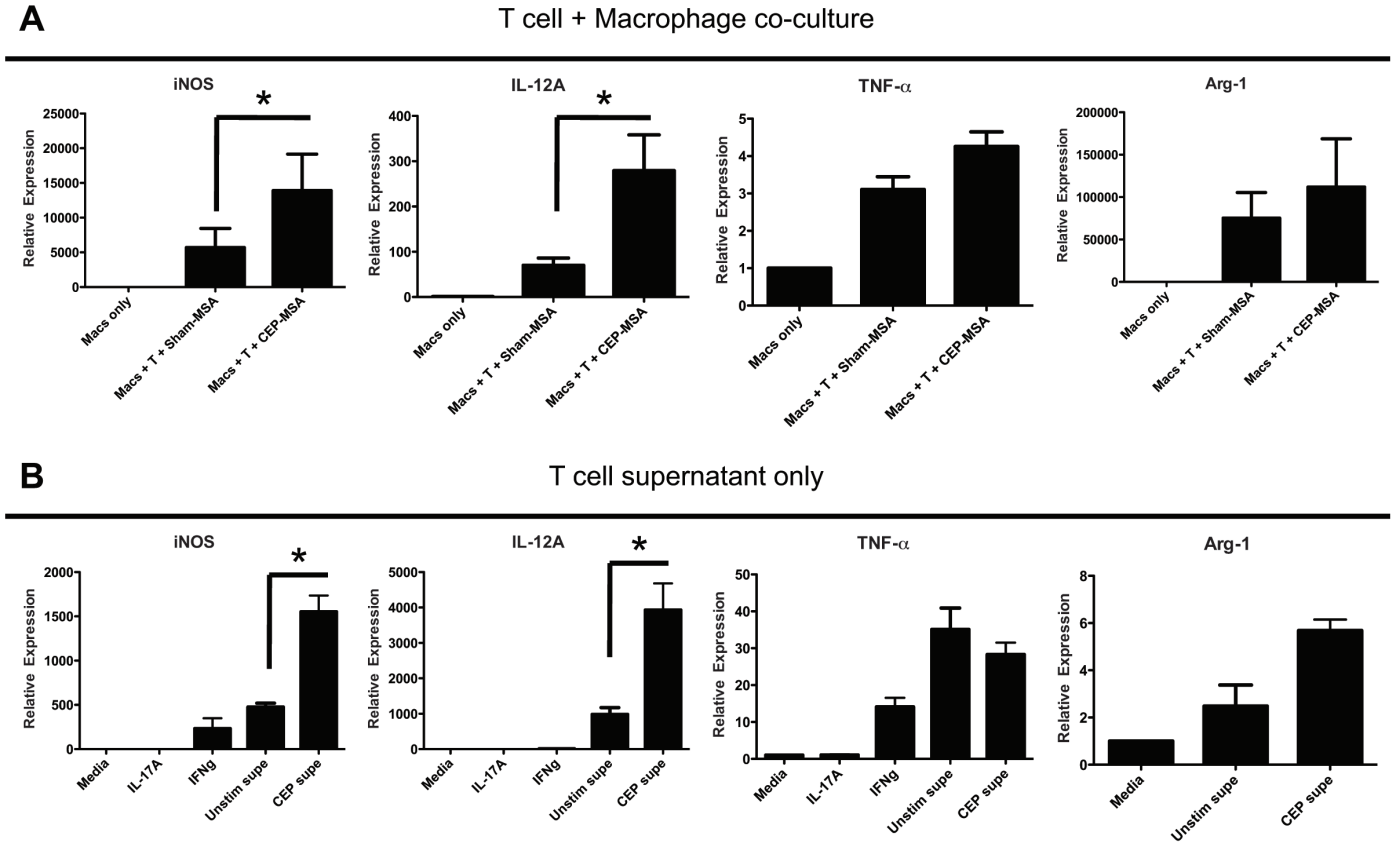
CEP-specific T cells induce M1 macrophage polarization *in* *vitro*. (**A**) Splenocytes from CEP-immunized mice were stimulated *in* *vitro* with CEP-MSA (or left unstimulated). At day 4 of stimulation, live cells were isolated and used for co-culture with primary macrophages for 24 hrs. After co-culture period, macrophages were isolated, RNA was extracted and qPCR used for gene expression analysis. Each treatment is represented as relative expression (fold-expression over Macrophage only). IL-10 showed no CEP effect (data not shown). Data from at least 3 independent experiments were pooled for each analysis. Two-tailed Student’s t test was used for statistical analysis (* denotes p<0.05). (**B**) Supernatants (secreted factors) from CEP-stimulated or unstimulated splenocytes were used for primary macrophage stimulation for 4 hrs. Purified IL-17A (1 ng/ml) and purified IFN-γ (1 ng/ml) were used for control stimulations. Each treatment is represented as relative expression (fold-expression over macrophages stimulated in Media only). Data from at least 3 independent experiments were pooled for each analysis. ANOVA was used for statistical analysis (* denotes p<0.05).

Our combined data suggests that both macrophages and T cells are needed to generate CEP-mediated retinal degeneration. Activation or function of just one of the two cell types is not sufficient for disease development. M1 macrophages are the main effector cells at the site of injury [26]; we have not detected T cells of any kind within the outer retina, although some T cells are present in the choroid of CEP-immunized mice (data not shown). Macrophages need to be recruited to the retina for the pathology to ensue, even if CEP-specific T cell activation and antibody production are normal, as is the case in Ccr2-deficient mice ([26] and data not shown). On the other hand, T cell activation is required for pathology even if a normal monocyte/macrophage compartment is present, as is the case in RAG−/− mice [40] and T-bet ko mice (this study). Therefore, we addressed the issue of T cell-macrophage interactions by setting up a simplified co-culture system using CEP-specific T cells and “naïve” bone marrow-derived macrophages (BMDM). As shown in **Fig. 7A**, CEP-specific T cells promote (in an antigen-specific manner) a distinct pattern of M1 polarization *in* *vitro* by inducing the expression of at least a subset of M1-related genes (iNOS and IL-12A but not TNF-α). The effect of CEP-specific T cells on M1 macrophage polarization does not require cell-cell contacts, as T cell-secreted factors (in purified CEP stimulation supernatants) induce a similar pattern of gene expression in BMDM (**Fig. 7B**). Notably, the supernatant effect is not simply due to the presence of IFN-γ or IL-17A in the supernatants (Fig. 5C), since similar concentrations of the purified cytokines fails to reproduce this effect. Importantly, expression of M2-related genes (such as Arg-1) is not significantly enhanced in the presence of T cells or their secreted factors (**Figure 7**).

### Proof-of-principle: Pharmacological Inhibition of T Cell Activation Prevents CEP-induced Retinal Pathology

**Figure 8:**
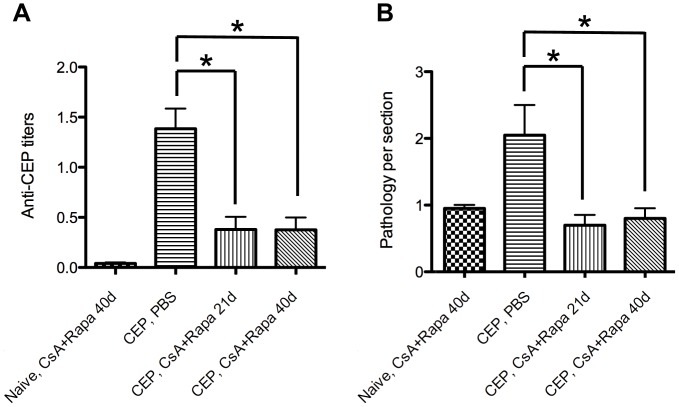
Proof of concept experiment showing that T cell inhibitory drugs Cyclosporin A and Rapamycin prevent CEP-induced retinal pathology. (**A**) WT BALB/c mice were immunized with CEP-MSA and treated every day (starting at day of immunization) with combined CsA+Rapa for either 21 or 40 days p.i. Control PBS injections were also performed. Serum was isolated at day 47 p.i. and anti-CEP titers were evaluated by ELISA. (**B**) Eyes were harvested at day 60 p.i. Retinal pathology scores for the indicated groups are shown (n = 3). ANOVA was used for statistical analysis (* denotes p<0.05). Results are representative of two independent experiments.

One of the ultimate goals of our research program is to learn the basic mechanisms involved in the AMD disease process so that we can attempt to translate such information into meaningful therapeutic strategies in the future. While immunotherapy has been proposed as an alternative for the treatment of AMD [16], there is no current published data formally proving the efficacy of this approach. As a proof-of-concept, we treated CEP-immunized mice with a combination drug therapy aimed at suppressing T cell responses [45]. In general, T cells require two signals for complete activation: T cell receptor (TCR) signaling and co-stimulation. Cyclosporine A (CsA) is an inhibitor of calcineurin, a phosphatase downstream of TCR signaling required for activation of NFAT and IL-2 production. Rapamycin (Rapa, also known as Sirolimus) inhibits the mTOR pathway, which is downstream of co-stimulatory molecules (such as CD28) and cytokine receptors. Treatment of CEP-immunized BALB/c mice with both CsA and Rapa lead to two important results: downregulation of anti-CEP titers (**Fig. 8A**) and prevention of CEP-induced retinal pathology (**Fig. 8B**).

## Discussion

**Figure 9:**
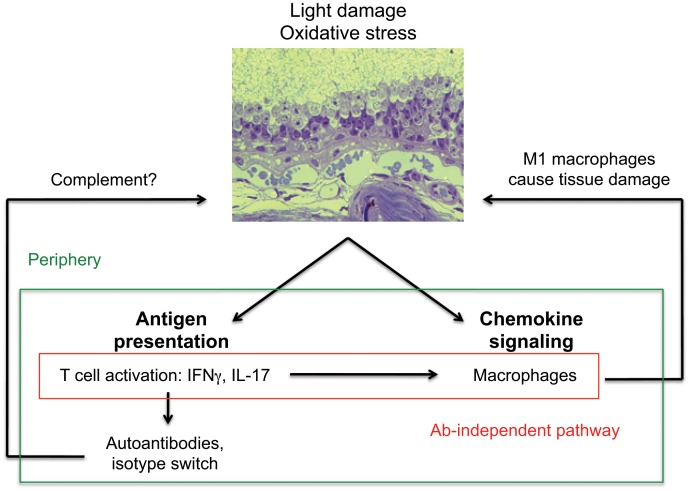
Working model for the cooperation of antigen-specific T cells and M1 macrophages in the development of AMD. Oxidative damage in the outer retina initiates a cascade of events that activate both innate and adaptive immune responses that ultimately lead to retinal damage.

This report provides several novel findings of great potential significance for the study of AMD pathogenesis, specifically, and chronic inflammatory diseases in general. We show that the innate and adaptive immune systems work in concert for disease development over extended periods of time, and we provide precise cellular and molecular mechanisms for this cooperation (**Fig. 9**). The lipid peroxidation product CEP serves as the functional link, capable of directly activating both M1 macrophages and antigen-specific T cells. CEP eye injections can also induce acute inflammation in the retina and human eyes have a distinctive CEP localization pattern in the retina, with diffuse presence within RPE of dry AMD patients. Our data help to clarify the role of antibodies in the CEP model of dry AMD by showing that CEP immunization leads to retinal lesions even in the absence of B cells. Moreover, CEP-specific T cells can induce M1 macrophage polarization *in* *vitro* and pharmacological suppression of T cell function prevents AMD-like pathology.

Previous evidence, including data from our lab, suggests that macrophages play a crucial role in the AMD disease process, but there is still controversy in this regard. It is possible that this discrepancy is partly due to the different stages of AMD progression: macrophages could have varied roles at the onset of dry AMD in response to oxidative damage, during the accumulation of drusen and during the transition from dry to wet AMD. It has been shown that iNOS-expressing macrophages infiltrate the Bruch’s membrane of AMD patients [46]. In addition, an intriguing pilot study by Chi-Chao Chan and colleagues showed that human AMD eyes had more M1 macrophages compared to normal eyes, which contained more M2 macrophages [47]. However, we cannot rule out the possibility that, in some instances, M2 or other types of macrophages (e.g. Mox?) can drive the dry AMD pathology. For instance, the important distinction between retinal resident microglia and macrophages recruited from the circulation awaits the development of microglia-specific markers. Regardless, the likely determining factor in macrophage polarization and AMD outcomes will be the patient-specific onset factors, which in our case is CEP. Our data on CEP tilting the balance toward M1 polarization *in vivo* and *in vitro,* in conjunction with the specific localization of CEP in the RPE of dry AMD eyes reported here, may explain the increased M1/M2 ratios observed in AMD patients [47]. Differences in drusen CEP patterns may also explain the focal nature of RPE lesions and geographic atrophy. Drusen with high CEP content could be hubs of inflammatory microenvironment. It was also interesting to find increased levels of CEP in cone photoreceptor (PR) cells of healthy donors, since the macula contains the highest concentration of cone cells in the eye. Is this a potential signal of propensity to develop AMD? The precursor for CEP, DHA, has been shown to be increased in cone versus rod PR cells in the gecko [48], but the specific content of CEP itself within PR subtypes of humans (and mice) requires further investigation. Unfortunately, we could not perform a similar CEP immunolabeling analysis in eyes from CEP-immunized and control mice due to high non-specific background using our mouse monoclonal antibody.

The first type of cells shown to directly respond to CEP were endothelial cells, which promote angiogenesis through TLR2 signaling [49]. Here, we show that two cell types relevant to dry AMD, macrophages and RPE cells, can directly respond to CEP. Doyle et al. [50] recently reported that macrophages could respond to CEP *in* *vitro,* priming the NLRP3 inflammasome through TLR2 ligation. While they report that NLRP3 is active in CEP-MSA-immunized mice, its role in macrophage differentiation is not known. Here we clearly show that CEP activation leads to an (M1) inflammatory response associated with tissue damage, which correlates with our published *in* *vivo* findings [26]. Whether there is a connection between macrophage polarization and inflammasome priming/activation in the context of AMD remains to be determined. Further characterization of the CEP receptor-mediated signaling events and transcriptional regulation in macrophages and RPE cells is under current experimentation in our lab.

What about the role of the adaptive immune system in AMD? Most of the evidence for adaptive immunity in AMD comes from the presence of anti-retinal autoantibodies in AMD patients [51]–[53], which is recapitulated in our CEP mouse model [40]. However, it is not clear if these antibodies (in humans) have deleterious or protective effects, or if they arise as secondary events and have no direct role in disease pathogenesis or progression. We have addressed this major question in our model by immunizing mice deficient in mature B cells *(μMT/*mice), which resulted in strong retinal lesions, indicating that the CEP-induced pathology is independent of antibodies. Because RAG-deficient mice do not develop AMD-like lesions [40], this result indicates that T cells are the major players within the adaptive immune system associated with disease in our model. This result, however, does not eliminate the possibility that antibodies may be involved in the AMD disease process. It may be possible for some anti-retinal antibodies to fix complement in the outer retina or to induce macrophages to damage the RPE. Conversely, some autoantibodies could actually serve a protective role. Regardless of their specific functions, autoantibodies represent useful targets for the development of AMD biomarkers. In this context, we suggest profiling autoantibody signatures, instead of single antibodies, to gain a better diagnostic and therapeutic picture.

To our knowledge, CEP-specific T cells are the second example of OSE-specific T cells in the literature. Malondialdehyde (MDA) is another lipid peroxidation product that serves as a marker of oxidative stress. MDA is formed upon peroxidation of polyunsaturated fatty acids present in phospholipids of low density lipoprotein (LDL), and has been associated with a number of oxidative stress-related diseases, such as atherosclerosis and AMD [29], [54]. Binder et al. [54] showed that immunization of mice with MDA-modified LDL (MDA-LDL) leads to an adaptive T cell-dependent immune response, with expansion of antigen-specific Th2 cells that mainly secrete IL-5. Therefore, these lipid modifications of self-proteins function as haptens and can actually dictate the T cell differentiation pathways (cytokine production profiles) of the responding cells. Recognition of oxidation by-products by T cells is an emerging paradigm in disease development, as it has also been shown that immune responses against oxidized lipoprotein is associated with atherosclerosis [55]. In the case of AMD, pro-inflammatory cytokine production by CEP-specific T cells contributes to the polarization of macrophages toward the M1 phenotype, providing a functional link between adaptive and innate immunity in the onset of disease.

T cell-produced cytokines can also integrate the antibody production to complete the response against CEP, as we have detected specific class isotype switching to IgG1. This switch is usually associated with Th2 (IL-4-mediated) responses, but we have not detected IL-4 production by CEP-specific T cells. In this regard, IFN-γ may not influence class switching but could certainly impact macrophage M1 polarization. This is supported by the lack of retinal macrophages in CEP-immunized T-bet ko mice. We attribute this result to the absence of Th1 cell differentiation and not to an intrinsic effect on T-bet ko macrophages, which have been reported to behave normally [56]. On the other hand, IL-17A may be more relevant to the antibody response, as there is some evidence that Th17 cells can promote IgG1 switching [57]. Even if IL-17A ko mice develop CEP-induced pathology, we do believe that IL-17 does play a significant role in AMD because IL-17 levels are elevated in AMD patients [34] and the *IL-17RC* promoter region is preferentially hypomethylated in AMD patients [58]. In fact, it has been shown in a retinal disease model that either Th1 or Th17 cells are capable of mediating disease [59]. Examining the role of IL-17-family cytokines (such as IL-17A and IL-17F) and their relationship with IFN-γ in AMD pathogenesis should clarify this issue in the future.

The use of pharmacological inhibition of T cell-mediated pathology in our model is a significant novel aspect of this work. We selected a strategy aimed at maximizing the possibility of T cell immunosuppression using both CsA and rapamycin. While we recognize that these two drugs may have other targets besides T cells, our interpretation of their effects in our model are based on complementary data, such as the results on B cell-deficient and T-bet ko mice. We have been careful in analyzing control mice and have seen no effect of this treatment on the retina. Specifically, control (CsA+rapa treated, without CEP immunization) mice remained healthy throughout the dosing period and their retinas looked normal. Similar results have been published in the context of the retina [60], at least in the case of i.p. rapamycin treatment (at higher doses of 3 mg/kg). To substantiate our approach, it is important to note that there are several ongoing clinical trials testing immunotherapy for AMD, including treatments that target TNF-α (one of the key effector molecules upregulated by CEP) and mTOR (the molecular target of rapamycin) [8]. Of course, there are important questions that need answers to complete our understanding of the macrophage and T cell pathways involved in the AMD disease process. What is the identity of the CEP receptor(s) in macrophages? Do macrophages reciprocally influence T cells? The precise mechanisms of antigen presentation of OSEs and TCR signaling dynamics are not known. Cloning OSE-specific TCR and generating transgenic mice (efforts currently underway in our lab) will certainly help address these issues. The subsequent analysis of such transgenic mice should provide valuable insight into the molecular mechanisms underlying initiation of immune-mediated AMD and could be used to test immunotherapies. Our proof-of-concept experiment with T cell suppression is a first step. Since we used a systemic, non-specific immunosuppressive approach, it would be interesting to develop antigen-specific therapies, such as depletion/suppression of CEP-specific T cells. Targeting the macrophage arm of the immune response could also have a beneficial outcome. For example, strategies that specifically prevent recruitment of blood-borne monocytes to the retina or that inhibit M1 polarization *in situ* may prove useful as AMD treatments. Our CEP model provides a great setting to test these and other protocols in future pre-clinical studies.

## Supporting Information

Figure S1 **CEP does not induce M2 or Mox gene expression in BALB/c macrophages** ***in*** ***vitro*** **.** Bone marrow-derived macrophages from BALB/c mice were stimulated for 4 hrs with CEP-MSA (100 µg/ml), Sham-MSA (100 µg/ml) or left untreated. RNA was isolated and qPCR was used for gene expression analysis. Each treatment is represented as relative-expression (i.e., fold-expression over reference group), where the control (untreated) sample served as the reference with a set value of 1. CEP did not influence expression of M2-related genes (Arg-1 and IL-10) or Mox-related genes (HO-1 and SXRN-1).
(TIF)

Figure S2 **CEP induces pro-inflammatory, but not angiogenic, gene expression in BALB/c macrophages** ***in*** ***vitro*** **.** Bone marrow-derived macrophages from BALB/c mice were stimulated for 4 hrs with CEP-MSA (100 µg/ml), Sham-MSA (100 µg/ml) or left untreated. RNA was isolated and qPCR was used for gene expression analysis. Each treatment is represented as relative-expression (i.e., fold-expression over reference group), where the control (untreated) sample served as the reference with a set value of 1. CEP specifically induced the expression of inflammation genes (IL-6 and KC) but had no effect on angiogenesis-related genes (Vegf-A and Vegf-B). As opposed to RPE cells, CEP did not induce Ccl2 expression in BMDM *in* *vitro*. Two-tailed Student’s t test was used for statistical analysis.
(TIF)

Figure S3 **CEP induces selective pro-inflammatory gene expression in RPE cells** ***in*** ***vivo*** **.** Intravitreal injections of CEP-MSA or Sham-MSA (2 µg total) were performed, RNA was isolated after 6 hrs from the RPE/choroid, followed by Taqman gene expression analysis (n = 5). While TNF-α expression was not upregulated upon CEP injections, KC levels were elevated in response to CEP. Mean values from one of two independent experiments are shown; error bars represent S.D. (*) denotes statistically significant differences (p<0.05) based on two-tailed Student’s t tests.
(TIF)

Figure S4 **Anti-CEP antibody production is maximized with CFA adjuvant.** WT BALB/c mice were immunized with CEP-MSA in the presence of either complete Freund’s adjuvant (CFA) or Alum. Anti-CEP titers were measured 40 days post-immunization (p.i.) (n = 5 per group). (*) denotes statistically significant differences (p<0.05) based on two-tailed Student’s t tests.
(TIF)
