# Supplementary figures and images for: T Cells and Macrophages Responding to Oxidative Damage Cooperate in Pathogenesis of a Mouse Model of Age-Related Macular Degeneration
Source: PLoS One. 2014 Feb 19;9(2):e88201. doi: 10.1371/journal.pone.0088201 (PMC3929609; doi:10.1371/journal.pone.0088201)

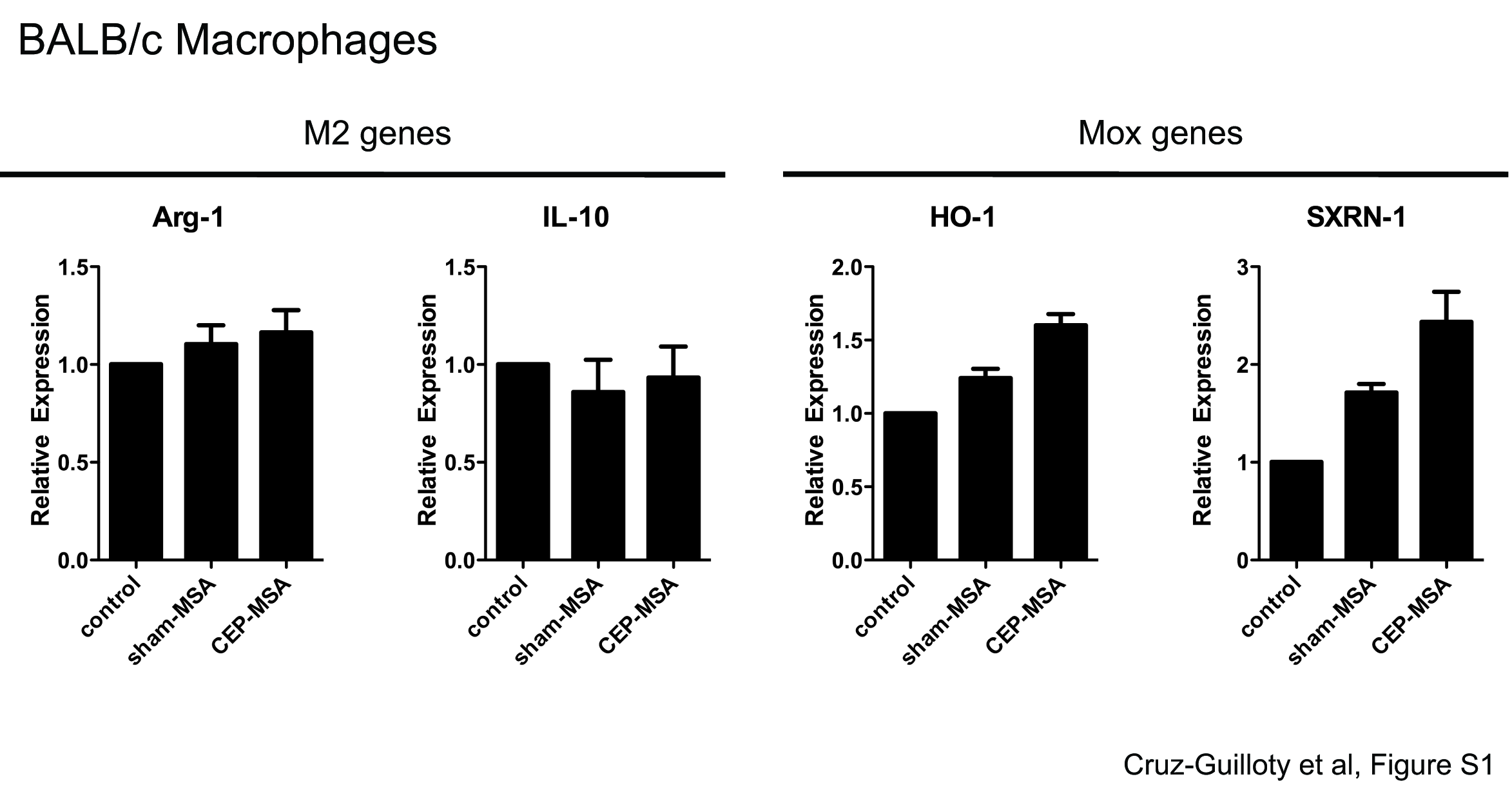

Supplement: Figure S1 — CEP does not induce M2 or Mox gene expression in BALB/c macrophages in vitro . Bone marrow-derived macrophages from BALB/c mice were stimulated for 4 hrs with CEP-MSA (100 µg/ml), Sham-MSA (100 µg/ml) or left untreated. RNA was isolated and qPCR was used for gene expression analysis. Each treatment is represented as relative-expression (i.e., fold-expression over reference group), where the control (untreated) sample served as the reference with a set value of 1. CEP did not influence expression of M2-related genes (Arg-1 and IL-10) or Mox-related genes (HO-1 and SXRN-1). (TIF) [file pone.0088201.s001.tif]

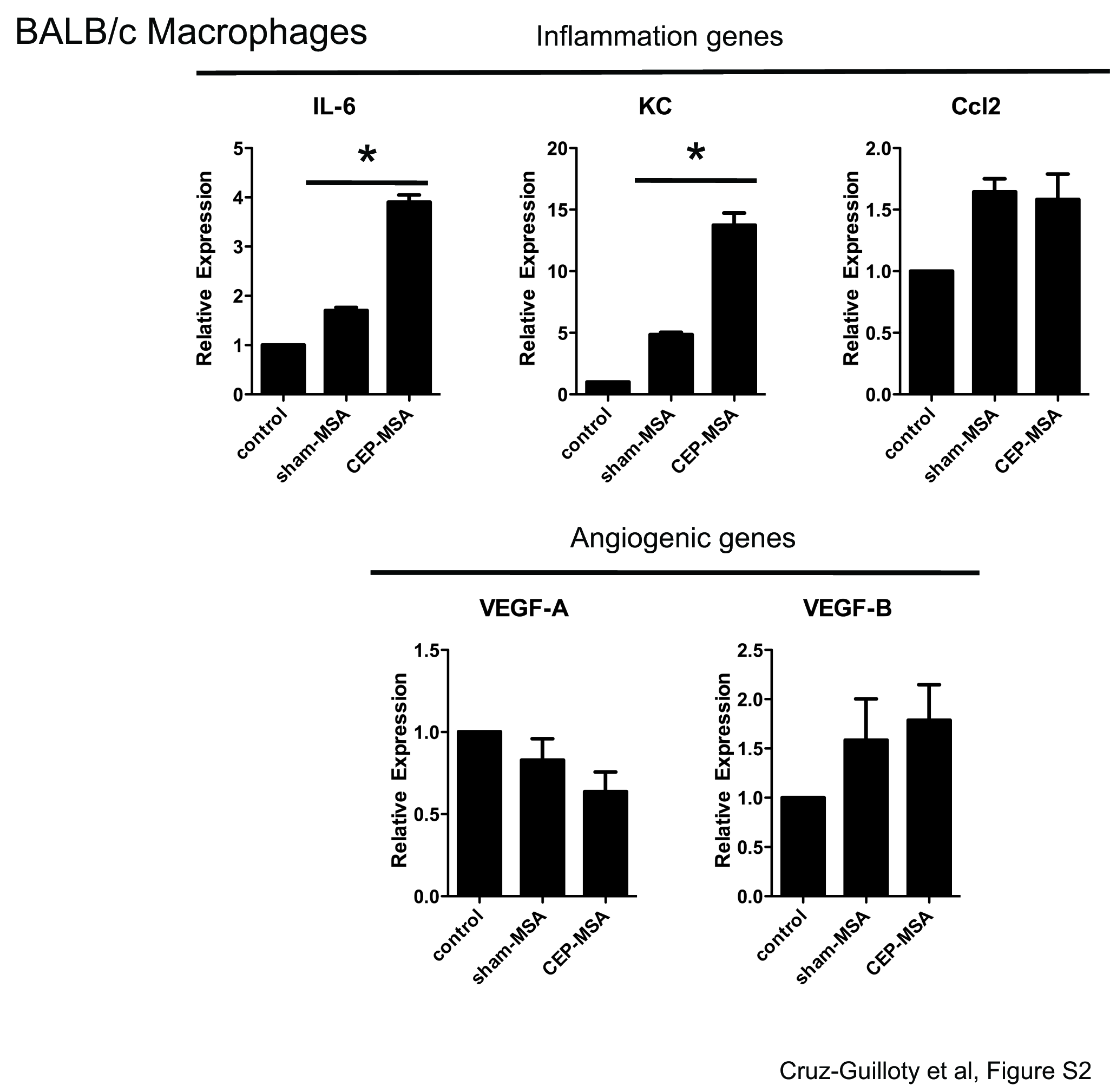

Supplement: Figure S2 — CEP induces pro-inflammatory, but not angiogenic, gene expression in BALB/c macrophages in vitro . Bone marrow-derived macrophages from BALB/c mice were stimulated for 4 hrs with CEP-MSA (100 µg/ml), Sham-MSA (100 µg/ml) or left untreated. RNA was isolated and qPCR was used for gene expression analysis. Each treatment is represented as relative-expression (i.e., fold-expression over reference group), where the control (untreated) sample served as the reference with a set value of 1. CEP specifically induced the expression of inflammation genes (IL-6 and KC) but had no effect on angiogenesis-related genes (Vegf-A and Vegf-B). As opposed to RPE cells, CEP did not induce Ccl2 expression in BMDM in vitro. Two-tailed Student’s t test was used for statistical analysis. (TIF) [file pone.0088201.s002.tif]

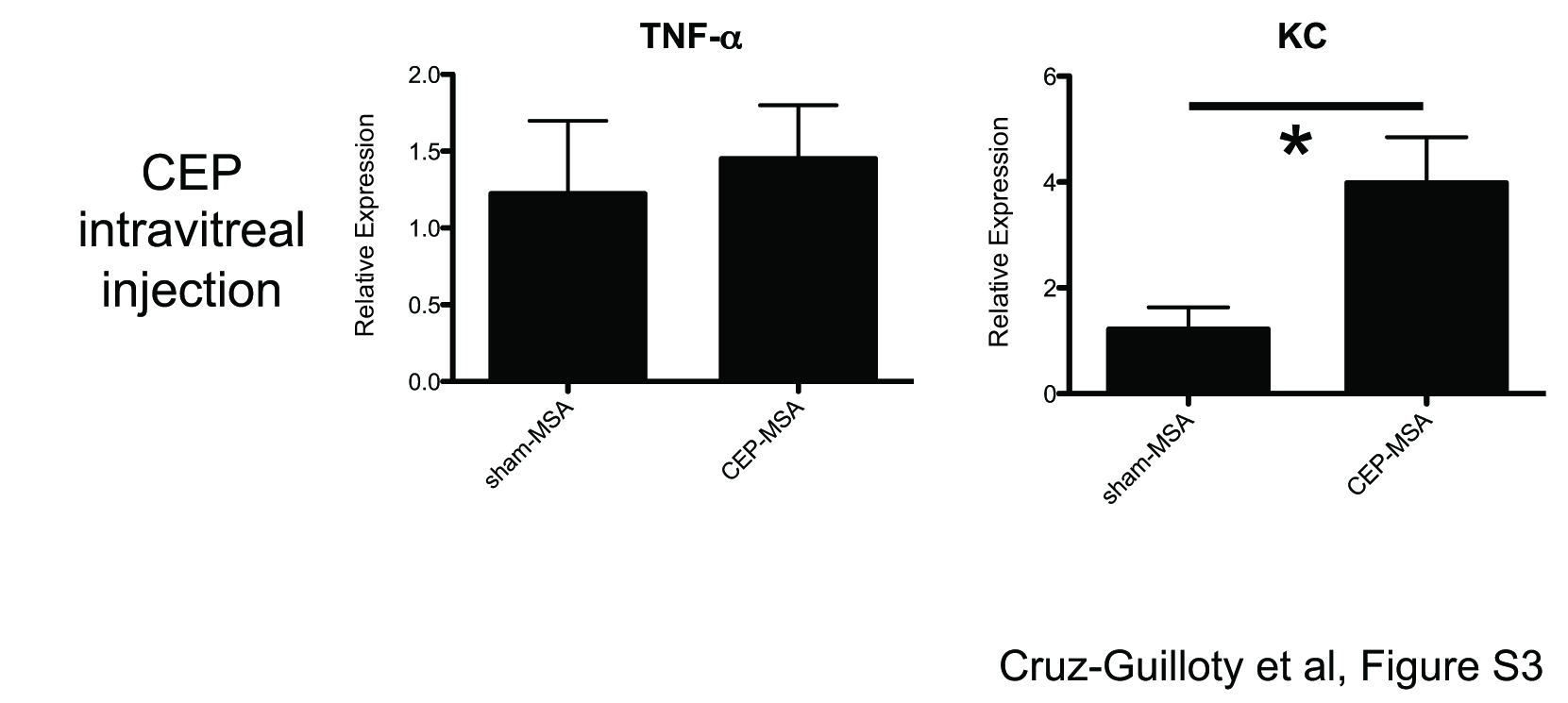

Supplement: Figure S3 — CEP induces selective pro-inflammatory gene expression in RPE cells in vivo . Intravitreal injections of CEP-MSA or Sham-MSA (2 µg total) were performed, RNA was isolated after 6 hrs from the RPE/choroid, followed by Taqman gene expression analysis (n = 5). While TNF-α expression was not upregulated upon CEP injections, KC levels were elevated in response to CEP. Mean values from one of two independent experiments are shown; error bars represent S.D. (*) denotes statistically significant differences (p<0.05) based on two-tailed Student’s t tests. (TIF) [file pone.0088201.s003.tif]

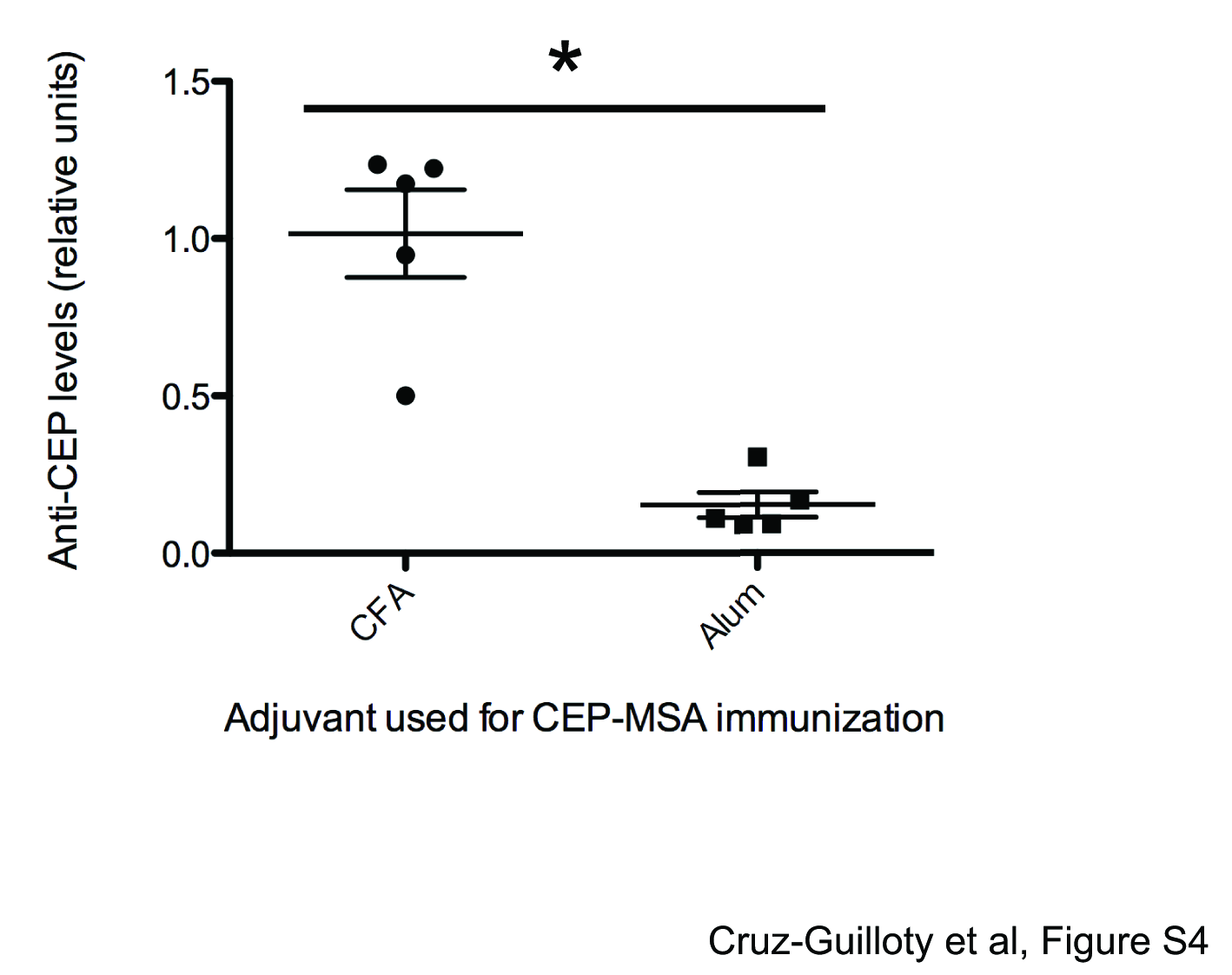

Supplement: Figure S4 — Anti-CEP antibody production is maximized with CFA adjuvant. WT BALB/c mice were immunized with CEP-MSA in the presence of either complete Freund’s adjuvant (CFA) or Alum. Anti-CEP titers were measured 40 days post-immunization (p.i.) (n = 5 per group). (*) denotes statistically significant differences (p<0.05) based on two-tailed Student’s t tests. (TIF) [file pone.0088201.s004.tif]
